# Possibility of SARS-CoV-2 Infection in the Metastatic Microenvironment of Cancer

**DOI:** 10.3390/cimb44010017

**Published:** 2022-01-05

**Authors:** Takuma Hayashi, Kenji Sano, Ikuo Konishi

**Affiliations:** 1 National Hospital Organization, Kyoto Medical Center, Kyoto 612-8555, Japan; ikuokonishi08@yahoo.co.jp; 2 START-Program, Japan Science and Technology Agency (JST), Tokyo 102-8666, Japan; 3 Shinsyu University Hospital, Matsumoto 390-8621, Japan; kenjisano12@yahoo.co.jp; 4 Graduate School of Medicine, Kyoto University, Kyoto 606-8501, Japan

**Keywords:** alveolar epithelial stem cells, ACE2, SARS-CoV-2, COVID-19, RBD of spike glycoprotein, metastatic microenvironment

## Abstract

According to a report from the World Health Organization (WHO), the mortality and disease severity induced by the severe acute respiratory syndrome coronavirus 2 (SARS-CoV-2) are significantly higher in cancer patients than those of individuals with no known condition. Common and cancer-specific risk factors might be involved in the mortality and severity rates observed in the coronavirus disease 2019 (COVID-19). Similarly, various factors might contribute to the aggravation of COVID-19 in patients with cancer. However, the factors involved in the aggravation of COVID-19 in cancer patients have not been fully investigated so far. The formation of metastases in other organs is common in cancer patients. Therefore, the present study investigated the association between lung metastatic lesion formation and SARS-CoV-2 infectivity. In the pulmonary micrometastatic niche of patients with ovarian cancer, alveolar epithelial stem-like cells were found adjacent to ovarian cancer. Moreover, angiotensin-converting enzyme 2, a host-side receptor for SARS-CoV-2, was expressed in these alveolar epithelial stem-like cells. Furthermore, the spike glycoprotein receptor-binding domain (RBD) of SARS-CoV-2 was bound to alveolar epithelial stem-like cells. Altogether, these data suggested that patients with cancer and pulmonary micrometastases are more susceptible to SARS-CoV-2. The prevention of de novo niche formation in metastatic diseases might constitute a new strategy for the clinical treatment of COVID-19 for patients with cancer.

## 1. Introduction

The United States and other countries have found it difficult to contain the coronavirus disease 2019 (COVID-19) pandemic due to the spread through respiratory droplets of the severe acute respiratory syndrome coronavirus 2 (SARS-CoV-2) and the inconsistent adherence to effective public health measures, including wearing masks and maintaining social distancing. According to the reports of the World Health Organization, the mortality rate of cancer patients infected with SARS-CoV-2 is 7.6%, which is fairly higher than the 1.4% mortality rate of individuals infected with SARS-CoV-2 without complications [1]. Among patients with cancer and COVID-19, the 30-day all-cause mortality is high, i.e., a mortality rate of 13.3%, and has been associated with general and cancer-specific risk factors [1,2]. Moreover, according to a report from the Japan Ministry of Health, Labor, and Welfare, as of August 2020, the severity rate of patients with solid cancer infected with SARS-CoV-2 was much higher than that of all patients infected with SARS-CoV-2 [3]. The reason why COVID-19 is more severe in cancer patients is not fully understood. However, immunity against the virus seems reduced in cancer patients receiving therapeutic anticancer agents [4].

Lung stem cells able to regenerate lung tissue are used in research on lung diseases, including cancer and infectious diseases. Furthermore, it has become possible to prepare a lung culture model using lung stem cells in the laboratory. However, the biological characteristics of human lung stem cells have not been clarified. Therefore, the development of a lung culture model, especially one modeling the most peripheral part of the lung where gas exchange occurs, has not progressed.

Human lung culture systems have been reported to model lung infections, including SARS-CoV-2 infections responsible for COVID-19-associated pneumonia [5,6]. Moreover, Kuo et al. succeeded in creating a human peripheral lung culture model using lung stem cells [5]. Epithelial cell adhesion molecule-positive alveolar epithelial type 2 (AT2) stem cells differentiate into AT2 progenitor cells that express angiotensin-converting enzyme 2 (ACE2). ACE2 is a receptor for SARS-CoV-2. In contrast, cytokeratin 5-positive lung stem cells differentiate into bronchial and/or bronchiolar epithelial progenitor cells. In other words, the AT2 progenitor cells used as human peripheral lung culture model express ACE2, a host cell receptor for SARS-CoV-2 [5]. Thus, SARS-CoV-2 can infect alveolar epithelial stem cells.

Previous clinical studies have shown that SARS-CoV-2 infection rates and COVID-19 severity are higher in cancer and in people with a history of cancer than they are in healthy individuals. The specific reasons for these observations have not been clarified. Previously, we have shown that, in lung metastases of ovarian cancer, alveolar epithelial cells adjacent to ovarian cancer cells transform into alveolar epithelial stem-like cells [7]. ACE2 is strongly expressed in alveolar epithelial stem-like cells [5]. The present study aims to confirm the expression of ACE2 in alveolar epithelial stem-like cells adjacent to ovarian cancer cells in lung metastases of ovarian cancer and to investigate the binding of SARS-CoV-2 spike glycoprotein to alveolar epithelial stem-like cells. We showed that ACE2 was indeed expressed in alveolar epithelial stem-like cells adjacent to ovarian cancer in the pulmonary micrometastatic niche. Furthermore, the receptor-binding domain (RBD) of SARS-CoV-2 spike glycoprotein bound to alveolar epithelial stem-like cells. Altogether, these data indicate that cancer patients with pulmonary micrometastases might be more susceptible to SARS-CoV-2. The prevention of de novo niche formation in metastatic diseases might constitute a new strategy for the clinical treatment of COVID-19 in patients with cancer.

## 2. Materials and Methods

### 2.1. Case Selection for Immunohistochemical Staining

To examine the biological and medical characteristics of the pulmonary micrometastatic niche, 4 cases with high-grade serous ovarian adenocarcinomas were selected from 69 primary epithelial ovarian cancers stained using an anti-human S100 calcium-binding protein A4 (S100A4) antibody. S100A4 is used as marker for ovarian cancer (Supplementary Table S1). Sixty-nine patients with ovarian carcinoma visited the Shinshu University Hospital (Matsumoto, Nagano, Japan) between 1994 and 2003. They underwent surgery followed by combination chemotherapy with taxane-based and platinum preparations. The Supplementary Materials show the detailed medical conditions of the patients.

### 2.2. Antibodies and Immunohistochemistry (IHC)

IHC staining of S100A4, cluster of differentiation 90 (CD90 or Thy1), ACE2, and the RBD of the SARS-CoV-2 spike glycoprotein was performed on tissue sections from pulmonary micrometastases of patients with high-grade serous ovarian cancer. Tumor tissue sections were incubated with the appropriate primary antibodies at 4 °C overnight. For the staining of the RBD of the SARS-CoV-2 spike glycoprotein, tumor tissue sections were incubated with 10 ng of recombinant RBD of the spike protein (Sino Biological Inc., Beijing, China) at 4 °C overnight. After this incubation, the sections were incubated with the mouse monoclonal antibody recognizing the RBD of SARS-CoV-2 spike glycoprotein at 4 °C overnight.

IHC-stained sections were visualized under a confocal microscope (Leica TCS SP8, Wetzlar, Germany) according to the manufacturer’s procedure. Photographs of the healthy alveoli and bronchioles areas (Bron.) as well as of metastases areas (Met.) were taken from tissue sections of lung metastases resected from patients with ovarian cancer, as shown in the result section. Then, the expression levels of each factor were calculated using fluorescent color. These IHC experiments on human tissue sections were performed using standard procedures at Shinshu University (Matsumoto, Nagano, Japan) and the National Hospital Organization Kyoto Medical Center (Kyoto, Kyoto, Japan) in accordance with the institutional guidelines (approval no. M192). The Supplementary Materials section contains the list of primary or secondary antibodies used in the experiments and the detailed materials and methods.

### 2.3. Ethical Approval and Consent to Participate

This study was reviewed and approved by the Central Ethics Review Board of the National Hospital Organization Headquarters in Japan (Tokyo, Japan) and Shinshu University (Nagano, Japan). The ethical approval was obtained on 17 August 2019 (approval number NHO H31-02). The authors attended educational lectures supervised by the Japanese Government on medical ethics in 2020 and 2021. The completion numbers for the authors are AP0000151756, AP0000151757, AP0000151769, and AP000351128. Consent to participate was needed as this work was considered clinical research. All subjects signed informed consent when they were briefed about the experiments and agreed with the contents of the study. The authors attended a seminar on the ethics of experimental research using human materials on 2 July 2020 and 20 July 2021 to become familiar with the importance and ethics of clinical experiments (National Hospital Organization Kyoto Medical Center and Shinshu University School of Medicine). The experiments with human materials performed in the present study were approved by the ethics committee (approval number KMC R02-0702).

Details of materials and methods are described in the Supplementary Materials.

## 3. Results

**Figure 1:**
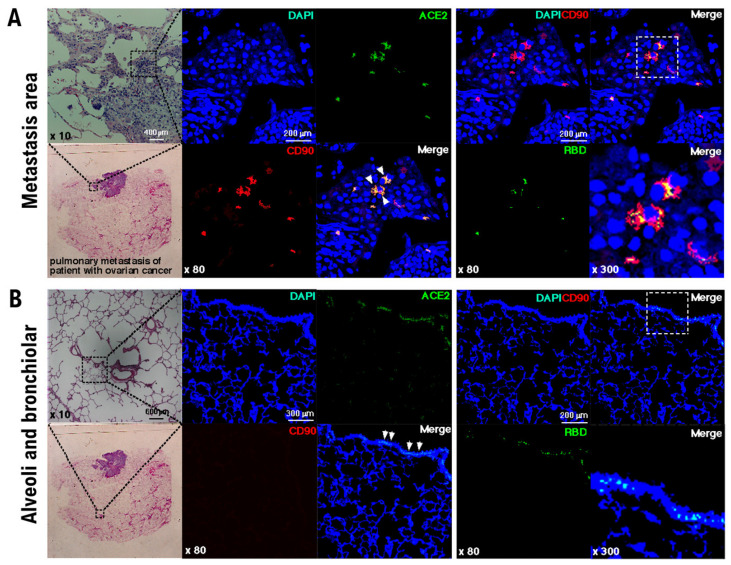
Binding of the RBD of the SARS-CoV-2 spike glycoprotein to the stem-like cells in normal neighboring alveolar epithelial cells. Immunohistochemical studies were performed using pulmonary metastatic tissue surgically excised from patients with high-grade serous ovarian carcinoma. The expression levels of CD90 and ACE2 and the binding of RBD in the normal alveolar and bronchiolar areas as well as in the metastases were investigated by pathological studies. In the photographs of the metastases (**A**) and normal alveolar and bronchiolar areas (**B**), the expression of each factor and binding activity are indicated by fluorescent color. Human ACE2-positive (green) and CD90-positive (red) stem-like cells, indicated by white arrowheads in human normal neighboring alveolar epithelial cells, were found in pulmonary micrometastases. Human CD90-positive cells (red) were not detected in the metastatic colonies of human serous ovarian carcinoma. Immunohistochemical studies were performed using an antibody recognizing human ACE2 (green), a monoclonal antibody detecting the spike glycoprotein of SARS-CoV-2 (green), and an antibody specific for human CD90 (red), which is a biomarker for stem-like cells. The binding of the RBD of SARS-CoV-2 spike glycoprotein (green) to CD90-positive (red) alveolar epithelial stem-like cells was observed and is indicated in yellow. Anti-human CD90 (Abcam ab133350), anti-human ACE2 (ORIGENE, Rockville, MD, USA), and anti-spike glycoprotein of SARS-CoV-2 (GeneTex, Inc., Irvine, CA, USA) antibodies, as well as recombinant spike glycoprotein of SARS-CoV-2 protein (BioVision, Milpitas, CA, USA), were used. The experiments were performed five times with similar results.

Niches promoting metastatic colonization have been previously investigated using models with human-in-mouse ovarian cancer xenograft in immunodeficient mice. For example, CD34-positive lineage ovarian cancer stem-like cells sorted using the side population procedure were injected into the mammary fat pads of BALB/c *nu/nu* mice [8]. CD90, also known as Thy1, is used as a marker for several stem cells [9]. S100 calcium-binding protein A4 (S100A4), a member of the S100 calcium-binding protein family secreted by ovarian cancer cells, supports tumorigenesis by stimulating angiogenesis [9] (Supplementary Table S1). Pathological examinations have shown the existence of S100A4-negative and CD90-positive stem-like cells in vimentin-positive normal neighboring alveolar epithelial cells [9]. Similar to this previous observation, we found that the initialization of mimicry represented incomplete differentiation of normal alveolar epithelial cells toward the stem-like lineage in pulmonary micrometastases of patients with ovarian cancer (Figure 1A).

ACE2, a host-side receptor for SARS-CoV-2, expressed in CD90-positive alveolar epithelial stem-like cells in the pulmonary metastatic niches of patients with high-grade serous ovarian cancer, is essential (Figure 1A and Table 1). Furthermore, histopathological analyses showed that the RBD of the SARS-CoV-2 spike glycoprotein bound to ACE2-expressing CD90-positive alveolar epithelial stem-like cells (Figure 1A and Table 1). Based on these findings, SARS-CoV-2 is deemed to infect the alveolar epithelial stem-like cells in pulmonary micrometastases of patients with ovarian cancer.

The pathological examination with an anti-human CD90 monoclonal antibody revealed that CD90 was not expressed in the alveolar and bronchiolar epithelial cells (Figure 1B). However, analyses with an anti-human ACE2 monoclonal antibody showed that bronchiolar epithelial cells expressed ACE2 (Figure 1B, Table 1). Therefore, the binding of SARS-CoV-2 RBD to the ACE2-positive bronchiolar epithelial cells was confirmed in the normal tissue section (Figure 1B).

**Figure 2:**
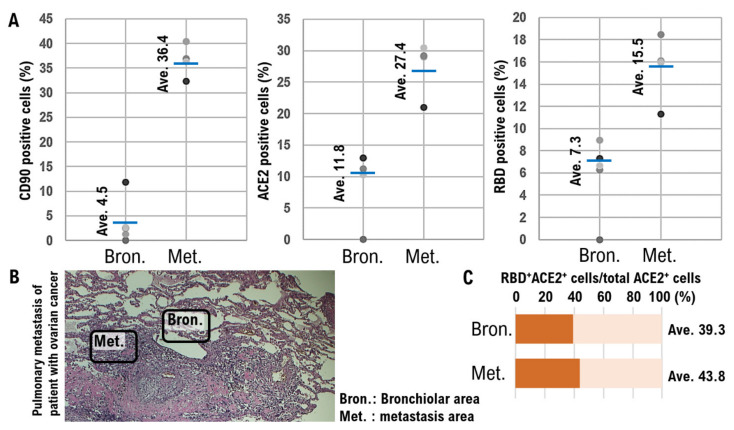
Quantification of immunofluorescence colocalization in imaging for CD90 and ACE2 expression levels and RBD binding. A quantitative analysis was conducted using ImageJ Version 1.53 m, a public domain software for image analysis (NIH ImageJ, Bethesda, MD, USA). The CD90 and ACE2 expression levels and the binding of RBD in normal alveolar and bronchiolar areas (Bron.) and metastases areas (Met.) were investigated through pathological studies. (**A**) The ratios of the number of target protein-positive cells to the total number of cells are shown in the dispersion diagram. (**B**) Photographs of the normal alveoli and bronchioles areas (Bron.) and metastases areas (Met.) from a tissue section of lung metastases resected from a patient with ovarian cancer. The expression levels of each factor were determined by measuring fluorescence intensities. (**C**) The proportion of bronchiolar epithelial cells associated with RBD of SARS-CoV-2 in normal alveoli and bronchiolar regions is indicated as the ratio of RBD-positive cells to total cell count.

In the photographs of the normal alveolar and bronchiolar areas as well as the metastases areas, the expression levels of each factor were determined by measuring the fluorescence intensity. Moreover, the ratios of CD90-positive, ACE2-positive, and RBD-positive cells were determined in these areas. Quantitative analysis showed that the average ratio of CD90-positive cells was higher in the metastatic areas (36.4%) than that in the normal alveoli and bronchioles areas (4.5%) (Figure 2A, Table 1, Supplementary Figure S1). Additionally, the average proportion of ACE2-positive cells in the normal alveolar and bronchiolar areas was 11.8%, and that in the metastatic areas was 27.4% (Figure 2A, Table 1, Supplementary Figure S2). The average ratio of bronchiolar epithelial cells binding to SARS-CoV-2 RBD in normal alveolar and bronchiolar areas was 7.3%, whereas it reached 15.5% in the metastatic areas (Figure 2A, Table 1). Figure 2B shows the normal alveoli and bronchioles areas and metastases areas.

The ratio of ACE2 and RBD double-positive cells to the total number of ACE2-positive cells was 39.3% in the normal alveolar and bronchiolar areas and 43.8% in the metastatic areas (Figure 2C), suggesting that the binding property of RBD to ACE2-positive cells did not significantly change between normal alveolar and bronchiolar areas and the metastatic areas.

Previous studies have demonstrated that, in the pulmonary micrometastatic niche of other cancer types, the reprogramming of mimicry probably represents the incomplete differentiation of normal alveolar epithelial cells into stem-like lineages [7,10]. Presumably, the pulmonary micrometastatic niche is a target for SARS-CoV-2 infection.

## 4. Discussion

Previous clinical studies have shown that SARS-CoV-2 infection rates and COVID-19 severity rates are higher in cancer patients and in people with a history of cancer than in healthy individuals. The specific reasons for these observations have not been clarified. Previously, we showed that, in lung metastases of ovarian cancer, alveolar epithelial cells adjacent to ovarian cancer cells transform into alveolar epithelial stem-like cells [7]. Alveolar epithelial stem-like cells have been reported to strongly express ACE2 [5]. The present work aimed to confirm the expression of ACE2 in alveolar epithelial stem-like cells adjacent to ovarian cancer cells in lung metastases of ovarian cancer and to investigate the binding of the SARS-CoV-2 spike glycoprotein to alveolar epithelial stem-like cells. Our IHC analyses showed the expression of ACE2 in alveolar epithelial stem-like cells adjacent to ovarian cancer cells in lung metastases of ovarian cancer. We also demonstrated the binding of SARS-CoV-2 spike glycoprotein to alveolar epithelial stem-like cells.

A high 30-day all-cause mortality has been reported in patients with cancer and COVID-19. Various factors contributing to COVID-19 severity in cancer patients have been identified. A previous report has shown that SARS-CoV-2 infects alveolar and bronchiolar epithelial cells [11]. Within the pulmonary metastatic niche, alveolar epithelial cells adjacent to metastatic cancer cells are differentiated into alveolar epithelial stem-like cells. Our experiments showed that, although the expression of ACE2 was not strong in normal alveolar epithelial cells, ACE2 was clearly expressed in alveolar epithelial stem-like cells. We also observed the binding of the alveolar epithelial stem-like cells to the RBD of the SARS-CoV-2 spike glycoprotein. Therefore, SARS-CoV-2 infection of stem cells and/or epithelial progenitor cells present in the metastatic niche in patients with cancer is likely a factor contributing to COVID-19 severity.

We examined the environmental niche of lung metastases of ovarian cancer. However, the infection rate of SARS-CoV-2 and the severity of COVID-19 also increase in patients with other cancer types. The results showing that ovarian cancer cells form a metastatic niche near the alveolar stem cells are reminiscent of a previous finding demonstrating that prostate cancer cells metastasizing to the bone settle near the stem cells in the bone marrow, promoting the development of a metastatic environment that supports tumor growth [12]. A recent report also described cancer-associated parenchymal cells that show stem-cell-like characteristics, the expression of lung progenitor markers, multilineage differentiation potential, and self-renewal activity [13].

To obtain accurate histopathological information of positive or negative SARS-CoV-2 infection in tissues from COVID-19 cancer patients, histopathological experiments with metastatic tissues of cancer patients infected with SARS-CoV-2 or with COVID-19 symptoms must be performed. Thus far, treatment aimed at reducing lung metastases has been limited to surgical treatment. However, in clinical practice, lung metastases have been reduced using immune checkpoint inhibitors and/or poly ADP-ribose polymerase inhibitors. Furthermore, the efficacy of the anti-S100A4 antibody drug in suppressing the metastatic ability of malignant tumors in other organs, including ovarian cancer, has been investigated [9,14,15].

A longer follow-up is needed to better understand the effect of COVID-19 on the treatment outcomes of patients with cancer, including on the ability to continue specific cancer treatments. In such a significant intersection of cancer medicine and infectious diseases, the prevention of de novo niche formation of metastatic disease might constitute a novel strategy for the clinical treatment of COVID-19.

## 5. Conclusions

Previous clinical studies have shown that SARS-CoV-2 infection rates and COVID-19 severity rates are higher in cancer patients currently being treated and people with a history of cancer than in healthy individuals. The specific reasons for the high SARS-CoV-2 infection rate and COVID-19 aggravation rate in cancer patients have not been clarified. Infection of SARS-CoV-2 into the niche of metastatic lesions in cancer patients may be one of the reasons for the higher rate of SARS-CoV-2 infection and COVID-19 severity compared to healthy individuals.

## Figures and Tables

**Table 1:** Characteristics of patients with ovarian cancer and lung metastases as well as CD90 and ACE2 expression in the metastasis areas and the alveolar and bronchiolar areas.

**Patient No.**	**Age Range**	**Age at Surgery (Years)**	**Histological Type**	**FIGO Stage**	**Grade**	**No. of Lung Metastatic Lesions**	**Pulmonary Metastatic Niche**		**Vital Status**
							CD90* (%)	ACE2* (%)	
1	40 s	40–45	HG serous	IVA	3	Single	36.43	27.42	Alive
2	50 s	50–55	HG serous	IVA	3	Single	33.87	18.93	Alive
3	50 s	50–55	HG serous	IVA	3	Multiple	38.32	29.38	Deceased
4	40 s	45–50	HG serous	IVB	3	Multiple	32.67	28.05	Alive
Normal alveolar and bronchiolar areas									
**Patient No.**		**Normal Alveoli**				**Normal Bronchioles**			
		CD90* (%)		ACE2* (%)		CD90* (%)		ACE2* (%)	
1		4.53	11.82	3.23	20.67	4.53	11.82	3.23	20.67
2		3.91	12.57	3.18	21.46	3.91	12.57	3.18	21.46
3		4.34	12.71	3.45	22.05	4.34	12.71	3.45	22.05
4		4.08	13.43	2.98	21.92	4.08	13.43	2.98	21.92

FIGO stage, the FIGO (International Federation of Gynecology and Obstetrics) staging system is commonly used for cancers of the female reproductive organs. High grade (HG) serous, high-grade serous ovarian adenocarcinoma. CD90*, proportion of CD90-positive alveolar epithelial stem-like cells in pulmonary metastatic niches, normal alveoli, and bronchioles assessed by immunohistochemical experiments using anti-human CD90 monoclonal antibody. ACE2*, proportion of ACE2-positive alveolar epithelial stem-like cells in pulmonary metastatic niches, normal alveoli, and bronchioles assessed by immunohistochemical experiments using anti-human ACE2 monoclonal antibodies. The expression levels of each factor were determined by measuring the fluorescence intensities. Percentages are the ratio of CD90 or ACE2-positive cells to the total cell counts.

## Data Availability

This manuscript is an editorial and does not contain research data. Therefore, there are no research data or information to be published or opened.

## Supplementary Materials

The following supporting information can be downloaded at: https://www.mdpi.com/article/10.3390/cimb44010017/s1. Table S1: Ovarian cancer cells express S100A4. In particular, in the case of high grade serous ovarian cancer, S100A4 is strongly expressed; Figure S1: IHC experiments with anti-human ACE2 monoclonal antibody do not provide the medical evidence, which demonstrate CD90 expressions in any cells of respiratory tissues, i.e., cells making up nasopharynx, bronchus, and lung tissues. −, 0–10% positive cells; +, 10–50% positive cells, more than 50% positive cells; Figure S2: IHC experiments with anti-human ACE2 monoclonal antibody demonstrate that in case of ciliated cells (ciliary rootlets) of nasopharynx and bronchus, ACE2 is markedly expressed. However, lung cells i.e., alveolar cells and macrophages unclearly express ACE2. −, 0–10% positive cells; +, 10–50% positive cells, more than 50% positive cells.
