# Supplementary material for: Possibility of SARS-CoV-2 Infection in the Metastatic Microenvironment of Cancer
Source: Curr Issues Mol Biol. 2022 Jan 5;44(1):233–41. doi: 10.3390/cimb44010017 (PMC8929006; doi:10.3390/cimb44010017)
Supplement: Supplementary file 1 [file cimb-44-00017-s001.zip › cimb-1515939-supplementary.pdf]

Manuscript ID: cimb-1515939

Supplementary Materials for

Possibility of SARS-CoV-2 infection in metastatic microenvironment of cancer

Takuma Hayashi, Kenji Sano, Ikuo Konishi

Materials and Methods

1. Antibodies

List of antibodies, which were used as first monoclonal antibody or secondary antibody in our immunohistochemistry research experiments, is shown below.

Antibodies

Antibody	Company	Catalogue No.	Clonal (Clone)	Specificity	Dilution
S100A4	Abcam	ab124805	Rabbit monoclonal EPR2761(2)	Human	1:200 (IHC)
CD90	Abcam	ab133350	Rabbit monoclonal EPR3133	Human	1:200 (IHC)
ACE2	ORIGENE	CF803844	Mouse monoclonal 4C5	Human	1:150 (IHC)
RBD of Spike	GeneTex	GNT-9366-04	Mouse monoclonal 1A9 (GNT936604)	SARS-CoV-2	1:200 (IHC)
Anti-mouse IgG Alexa Fluor® 488	Invitrogen	A32723	Goat / IgG	Mouse IgG	1:100
Anti-Rabbit IgG Alexa Fluor® 546	Invitrogen	A-11035	Goat / IgG	Rabbit IgG	1:200

2. Case selection for immunohistochemical staining

Cases were selected from total of 69 primary epithelial ovarian cancers for immunohistochemical analysis. Sixty-nine consecutive patients with ovarian carcinoma visited Shinshu University Hospital between 1994 and 2003 and underwent surgery followed by cisplatin-based chemotherapy. The follow-up period ranged from 3 to 160 months (median: 76 months). According to the International Federation of Gynecology and Obstetrics (FIGO) classification, 33 carcinomas were classified as Stage I, 10 were classified as Stage II, 21 were classified as Stage III and 5 were classified as Stage IV. Ovarian epithelial cancers are classified into serous, mucinous, endometrioid, clear cell, transitional cell, squamous cell, mixed epithelial and undifferentiated categories depending on histomorphologic features[1]. Histologically, 25 were serous, 5 were mucinous, 22 were clear cell and 17 were endometrioid adenocarcinomas (Supplementary Table S1). In 24 of the 69 cases, the specimens of peritoneal dissemination were available and also examined for immunohistochemistry. The expression of S100A4 in the nucleus and cytoplasm in all 69 cases was examined by immunohistochemical staining (Supplementary Table S1). Serous ovarian cancer has a lower 5-year survival rate than other histological types (Supplementary Table S1). Of the five cases at stage IV, four cases of high grade serous ovarian carcinoma were available for lung metastases and were also examined for immunohistochemistry. Each tissue sample was used with the approval of the Ethics Committee of Shinshu University School of Medicine (approval no. M-19-305).

3. Immunohistochemistry (IHC)

IHC staining for CD90(Thy1), S100A4, ACE2, and RBD of spike glycoprotein of SARS-CoV-2 was performed on tissue sections of ovarian cancers. Antibodies for CD90(Thy1) (ab133350), S100A4 (ab124805) were purchased from Abcam Inc. (Cambridge, UK). RBD of spike glycoprotein was purchased from GeneTex Inc. (Irvine CA USA). DAPI Mounting Medium was purchased from VECTOR LABORATORIES, Inc. (Burlingame, CA). IHC was performed using normal methods with the primary antibody and second antibody conjugated with immunofluorescence as described previously. Briefly, one representative 5-µm-thick tissue section was cut from a paraffin-embedded sample derived from patients with ovarian cancer. Human lung tissue array for examination with normal lung tissues[1] (Cat.# BC04002b, US Biomax Inc. Rockville, MD, USA) was also used for IHC examination. To examine the expression levels of target molecules, we performed immunofluorescence experiments for CD90(Thy1), S100A4 on paraffin- embedded tissues derived from patients with ovarian cancer. Tumor tissue sections were then incubated with the appropriate primary antibodies at 4°C overnight. We used rabbit monoclonal antibodies to S100A4 (1:200), a rabbit monoclonal antibody to CD90(Thy1) (1:200), a mouse monoclonal antibody to human ACE2 (1:150), and RBD of spike glycoprotein of SARS-CoV-2 (1:200) as the primary antibody. Tumor tissue sections were incubated with 10 ng of RBD of spike recombinant protein (Sino Biological Inc. Beijing, China) at 4°C overnight. After incubating with RBD of spike recombinant protein, the reactions of tissue sections were incubated with the mouse monoclonal antibody to RBD of spike glycoprotein of SARS-CoV-2 at 4°C overnight. After being incubated with the secondary antibody, i.e., the Alexa Fluor® 488-conjugated anti-mouse IgG antibody or Alexa Fluor® 546-conjugated anti-rabbit IgG antibody (1:200; Invitrogen), sections were washed and cover slipped with mounting medium and 40,6-diamidino-2-phenylindole (DAPI) (Vectashield; Vector Laboratories) and then visualized under a confocal microscope (Leica TCS SP8, Wetzlar, Germany) according to the manufacturer's procedure. In the photographs of the normal alveoli and bronchioles areas (Bron.) and metastases areas (Met.) as shown in Figure 2B, the expression levels of each factor are calculated using fluorescent color. Normal rabbit or mouse antiserum was used as a negative control for the primary antibody. A quantitative analysis was performed by using Image J Version 1.53m, a public domain software for image analysis (NIH ImageJ, Bethesda, MD, USA). These experiments with human tumor tissues derived from patients with ovarian cancer were conducted at Shinshu University and National Hospital Organization Kyoto Medical Center in accordance with institutional guidelines (approval no. M192).

1. https://www.proteinatlas.org/learn/dictionary/pathology/ovarian+cancer

Supplementary Table S1 Ovarian cancer cells express S100A4. In particular, in the case of high grade serous ovarian cancer, S100A4 is strongly expressed.

Histological type	Total cases	Expression of S100A4						5-year survival rate
		Cytoplasmic staining			Nuclear staining			
		+	++	+++	+	++	+++	
Serous	25	0	3	22	11	6	8	59.9%
Mucinous	5	0	1	4	1	3	1	91.1%
Endometrioid	17	3	4	10	9	4	4	81.3%
Clear cell	22	2	5	15	13	5	4	77.5%

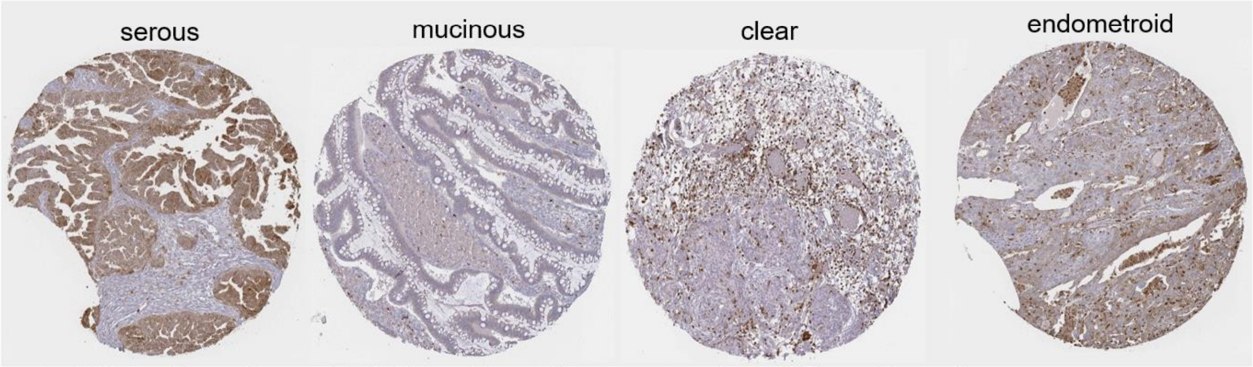

–, 0–10% positive cells; +, 10–50% positive cells; + +, more than 50% positive cells.

IHC with anti-human CD90 monoclonal antibody

Lung
Alveolar cells
Negative
Macrophages
Negative

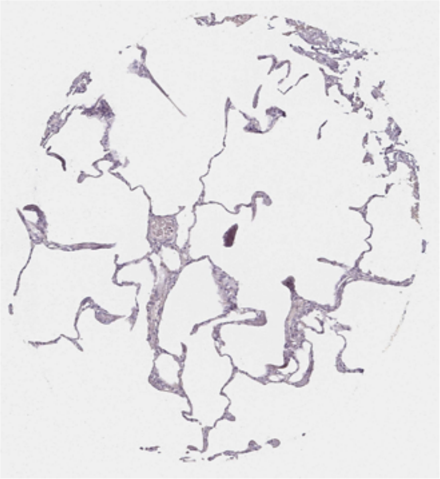

CD90 expression in Lung tissues	
Alveolar cells	–
macrophages	–

Supplementary Figure S1. IHC experiments with anti-human ACE2 monoclonal antibody do not provide the medical evidence, which demonstrate CD90 expressions in any cells of respiratory tissues, *i.e.*, cells making up nasopharynx, bronchus, and lung tissues. -, 0-10% positive cells; +, 10-50% positive cells, more than 50% positive cells.

IHC with anti-human ACE2 monoclonal antibody

Nasopharynx
Basal cells
Negative
Ciliated cells (cell body)
Negative
Ciliated cells (cilia axoneme)
Negative
Ciliated cells (ciliary rootlets)
Strong positive
Ciliated cells (tip of cilia)
Negative
Goblet cells
Negative

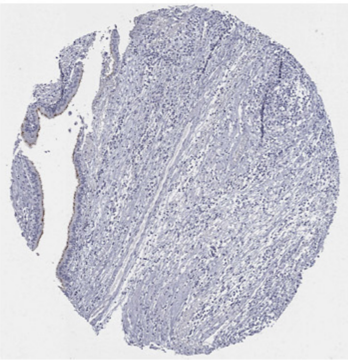

Bronchus
Basal cells
Negative
Ciliated cells (cell body)
Negative
Ciliated cells (cilia axoneme)
Negative
Ciliated cells (ciliary rootlets)
Strong positive
Ciliated cells (tip of cilia)
Negative
Goblet cells
Negative

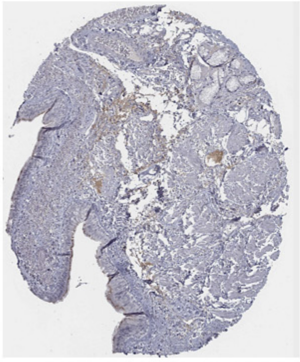

Lung
Alveolar cells
Negative
Macrophages
Negative

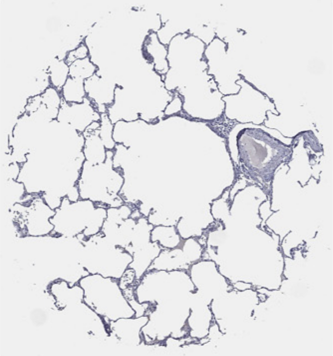

ACE2 expression			
Nasopharynx		Bronchus	
Basal cells	−	Basal cells	−
Ciliated cells (cell body)	−	Ciliated cells (cell body)	−
Ciliated cells (cilia axoneme)	−	Ciliated cells (cilia axoneme)	−
Ciliated cells (ciliary rootlets)	++	Ciliated cells (ciliary rootlets)	++
Ciliated cells (tip of cilia)	−	Ciliated cells (tip of cilia)	−
Goblet cells	−	Goblet cells	−

ACE2 expression in Lung tissues	
Alveolar cells	−
macrophages	−

Supplementary Figure S2. IHC experiments with anti-human ACE2 monoclonal antibody demonstrate that in case of ciliated cells (ciliary rootlets) of nasopharynx and bronchus, ACE2 is markedly expressed. However, lung cells i.e., alveolar cells and macrophages unclearly express ACE2. -, 0-10% positive cells; +, 10-50% positive cells, more than 50% positive cells.
